# Optimized EWT-Seq2Seq-LSTM with Attention Mechanism to Insulators Fault Prediction

**DOI:** 10.3390/s23063202

**Published:** 2023-03-17

**Authors:** Anne Carolina Rodrigues Klaar, Stefano Frizzo Stefenon, Laio Oriel Seman, Viviana Cocco Mariani, Leandro dos Santos Coelho

**Affiliations:** 1 Graduate Program in Education, University of Planalto Catarinense, Lages 88509-900, Brazil; 2 Digital Industry Center, Fondazione Bruno Kessler, 38123 Trento, Italy; 3 Department of Mathematics, Computer Science and Physics, University of Udine, 33100 Udine, Italy; 4 Department of Electrical Engineering, Federal University of Parana, Curitiba 81530-000, Brazil; 5 Graduate Program in Applied Computer Science, University of Vale do Itajai, Itajai 88302-901, Brazil; 6 Industrial and Systems Engineering Graduate Program, Pontifical Catholic University of Parana, Curitiba 80215-901, Brazil; 7 Mechanical Engineering Graduate Program, Pontifical Catholic University of Parana, Curitiba 80215-901, Brazil

**Keywords:** attention mechanism, empirical wavelet transform, fault prediction, insulators, long short-term memory, seasonal decomposition, time series forecasting

## Abstract

Insulators installed outdoors are vulnerable to the accumulation of contaminants on their surface, which raise their conductivity and increase leakage current until a flashover occurs. To improve the reliability of the electrical power system, it is possible to evaluate the development of the fault in relation to the increase in leakage current and thus predict whether a shutdown may occur. This paper proposes the use of empirical wavelet transform (EWT) to reduce the influence of non-representative variations and combines the attention mechanism with a long short-term memory (LSTM) recurrent network for prediction. The Optuna framework has been applied for hyperparameter optimization, resulting in a method called optimized EWT-Seq2Seq-LSTM with attention. The proposed model had a 10.17% lower mean square error (MSE) than the standard LSTM and a 5.36% lower MSE than the model without optimization, showing that the attention mechanism and hyperparameter optimization is a promising strategy.

## 1. Introduction

Insulators that are installed outdoors are exposed to environmental variations such as the accumulation of contaminants on their surface, which are mainly from industrial waste, salinity in coastal regions, or dust from unpaved roads [1]. These contaminants increase the surface conductivity of the network’s insulating components, causing higher leakage currents until a flashover occurs. The power grid is monitored through inspections of the electrical power system by specialized personnel, however, it is difficult to determine the specific location of failure when the faults are not visibly distinguishable [2].

Since there is little visual difference between faulty and non-faulty contaminated insulators, the classification of adverse conditions regarding this condition is a challenging task [3]. When there is a missing or partially broken insulator, this can be easily noticed, and finding the location of a fault in which the insulator is complete is more difficult; for this reason, specific types of equipment are used such as ultrasound detector, radio interference, ultraviolet camera, among others. One way to assess the impact of contamination on the grid insulation is the leakage current [4].

Although leakage current is a direct measurement method in which it is necessary to be connected to the electric potential, and this potential can be in high-voltage, this method is one of the most efficient to determine the supportability of an insulator to adverse conditions [5]. For this reason, the leakage current values from contaminated insulators will be used for the evaluation presented in this paper. Therefore, the data used are from a high-voltage laboratory experiment under controlled conditions.

The basic sequence-to-sequence (Seq2Seq) long short-term memory (LSTM) model for time series forecasting comprises an encoder LSTM that processes the input time series data and generates a context vector of fixed length, and a decoder LSTM that generates the forecasted values. This approach may not be optimal, however, when the time series data are lengthy or contain intricate patterns that are difficult to capture with a context vector of the specified length [6].

Attention mechanisms can circumvent these limitations by enabling the model to selectively concentrate on the most significant portions of the input time series data at each stage of decoding. In particular, the attention mechanism computes a set of attention weights for each input time step, indicating how much attention the model should pay to that time step when generating the output forecast [7].

By employing attention, the model can selectively focus on the most pertinent portions of the input time series data, such as the time steps that contain significant patterns or trends, and disregard the less pertinent portions of the data. This can enhance the model’s capacity to identify complex patterns in time series data and produce accurate forecasts [8].

On the other side, the empirical wavelet transform (EWT) is a mathematical tool that can be used to decompose time series data into various frequency bands with the goal of capturing the data’s essence and reducing the impact of noise or irrelevant information. EWT has been utilized in a variety of time series analysis applications, such as signal processing, image analysis, finance, and energy [9].

The EWT is founded on the concept of wavelets, which are functions that can represent data in both the time and frequency domains. In contrast to conventional Fourier analysis, which decomposes data into sinusoids with fixed frequencies, wavelets can represent data in terms of localized oscillations of varying frequencies and scales. This permits EWT to capture both short-term and long-term patterns in time series, making it more effective than techniques that solely rely on Fourier analysis to capture the essence of the data [10].

Thus, given the promising outcomes of the attention mechanism and the improvement in time series forecasting by the trend analysis with the EWT technique, this paper proposes a modified long short-term memory network model, named optimized EWT-Seq2Seq-LSTM with attention, wherein the Seq2Seq evaluation is based on two insulators.

The main contributions of this paper are:

- The use of two separate experiments (measuring the leakage current rise of contaminated high-voltage power grid insulators) for Seq2Seq evaluation enhances the generalizability of the analysis. This contribution addresses the need for robustness in forecasting models, as it ensures that the model can generalize well to unseen data.
- Model optimization using Optuna improves the selection of appropriate hyperparameters for the model, and the attention mechanism improves the model’s ability to predict forward values, thereby achieving an optimized structure. This contribution addresses the need for improved accuracy in forecasting models, as it ensures that the model is optimized to perform well on the given dataset.
- The use of empirical wavelet transform reduces signal variations that are not representative and maintains the trend variability, which is the focus of the failure prediction analysis evaluated in this paper. This contribution addresses the need for improved data-preprocessing techniques, as it ensures that the model is trained on meaningful features that capture the underlying patterns in the data.

The remainder of this paper is organized in the following way: Section 2 presents related works regarding time series analysis for fault prediction, and the proposed method is explained in Section 3. Section 4 is focused on the discussion of the results, and in Section 5, a conclusion and future directions of research are drawn.

## 2. Related Works

Fault prediction in insulators is a process of detecting potential faults or failures in electrical insulation systems before they occur. This helps prevent unplanned outages, equipment damage, and improves the overall power system reliability [11]. There are several techniques used for fault prediction in insulators, including a partial discharge measurement [12], thermal imaging [13], acoustic analysis [14], and online monitoring systems [15]. Using these techniques, it is possible to effectively predict faults in insulators and prevent them causing unplanned outages and equipment damage [16].

One means of monitoring that is rather promising is the evaluation of leakage current, which can be analyzed in the laboratory during an experiment, or after a controlled test is conducted [17]. An insulator leakage current refers to the flow of electrical current through an insulator that is intended to be an electrical barrier. This flow of current is usually due to defects or damages in the insulation system, which can result in partial discharge or the complete failure of the insulation [18].

By the controlling the current leakage levels, power system operators can improve the reliability, efficiency, and longevity of their equipment, reducing the risk of unplanned outages and equipment damage [19]. The leakage current prediction through time series analysis might be a means of helping in the monitoring of the electrical power system, which is the focus of the research presented in this paper.

### Time Series Forecasting Using LSTM with Attention

Time series forecasting is a common problem in many fields, including finance, healthcare, weather, transportation, and energy [20]. The appropriate forecasting of these series is important for making informed decisions and achieving optimal results. Learning to correctly predict time series can be challenging, but with the use of advanced machine learning models, such as LSTM, this has become more feasible [21].

LSTM is a recurrent neural network developed to handle complex time series. It is capable of handling long-term information and mitigating the forgetting of relevant information, making it more effective than other traditional machine learning techniques [22]. When training an LSTM model to forecast a time series, the model learns to identify patterns and trends in the series, including both short- and long-term trends. This enables the model to predict future values more accurately than if a simple forecasting technique, such as a moving average, were used [23].

In summary, using the LSTM for time series forecasting is a powerful and effective approach [20]. However, it is important to keep in mind that achieving reliable time series forecasting requires a combination of advanced machine learning approaches, reliable data, and technical skills to correctly adjust the model parameters. Several authors have presented promising works using the LSTM for time series forecasting [24].

A day-ahead residential load forecasting model based on feature engineering, pooling, and a hybrid model combining LSTM with a self-attention mechanism was proposed by Zang et al. [25]. The case studies were made on a dataset containing multiple residential users. The results showed the superiority of the proposed load forecasting model through comparison with other models. The volatility and intermittence of solar energy influence the accuracy of photovoltaic power prediction.

To improve the forecasting of this field, Qu et al. [26] proposed an attention-based long-term and short-term temporal neural network prediction model assembled using the convolutional neural network, LSTM, and an attention mechanism under the multiple relevant and target variables prediction pattern. The proposed model was superior when compared to classical models. Important parameters affecting the forecasting range of the model were analyzed, and suggestions were provided.

A univariate deep-LSTM-based stackable autoencoder model, fitted with a multi-stage attention mechanism for short-term load forecasting (15 and 30 min ahead) was proposed by Fazlipour et al. [27]. The model performance was evaluated by several tests employing realistic New England energy market data across three indices, demonstrating the superiority of the model and its strength in offline and online load forecasting. An attention mechanism was able to capture the temporal merit features lying in the LSTM unit.

Lin et al. [28] applied dual-stage attention based on LSTM for short-term load forecasting. Firstly, a feature attention-based encoder was constructed to calculate the correlation of the input features with the electricity load at each time step. Secondly, a temporal attention-based decoder was developed to mine the time dependencies. An LSTM model integrated these attention results and probabilistic predictions were obtained using a pinball loss function. The efficacy of the model was verified on the GEFCom2014 dataset, showing a higher generality capability compared to other state-of-the-art forecasting models.

An LSTM model enhanced by a dual-attention mechanism was proposed by Zhu et al. [29], which was inserted into the encoder–decoder to take into account the effects of different factors and time nodes to simultaneously analyze the characteristics of daily peak load to achieve more accurate prediction results. Experiments on a dataset of one city in eastern China showed that the proposed methodology provided promising results.

A deep learning-based interval prediction model combining fuzzy information granulation, attention mechanism, and LSTM was proposed by Li et al. [30] to predict the building energy consumption presenting future uncertainties in the form of ranges. A real-building dataset was used showing that attention-based LSTM provides better an interval prediction performance than conventional LSTM. Thus, the attention mechanism gains significant advantages by increasing the efficiency with which the model uses the information.

Meng et al. [31] applied the empirical wavelet transform to decompose the input features into multiple components after a hybrid attention mechanism-based LSTM was proposed as the forecasting model. The attention mechanism was used to dynamically investigate the importance of different input features, for example, the effect of renewable energy (wind and solar power generation) on the electricity price prediction. The model was validated on the datasets of the Danish electricity market with a high quantity of renewable energy, showing that the proposed model is superior to other hybrid models.

For simultaneously predicting both the active and reactive power, a new multi-task regression was proposed by Qin et al. [32] based on LSTM supported by the attention mechanism, to prevent performance deterioration, which was employed to accurately predict loads of a substation. The model was compared with other single-task load forecasting models achieving superior accuracy on both subtasks.

Dai et al. [33] proposed a novel combined short-term power load forecasting model, employing the weighted gray relational projection algorithm to distinguish the holidays and non-holidays, using the attention mechanism and extreme gradient boosting (XGBoost) to improve the LSTM model. The datasets from Singapore’s and Norway’s power markets were used to evaluate the prediction model by comparing it with six other models, outperforming in effectiveness, accuracy, and practicability.

Using an upgraded stacked gated recurrent unit–recurrent neural network for both uni-variate and multi-variate situations, Xia et al. [34] introduced a unique method for the prediction of renewable energy generation and electrical load. The suggested method is tested in two tests that anticipate the electricity load based on the historical energy consumption data and wind power generation utilizing a variety of meteorological conditions.

The ultrasound device is a piece of equipment used for the examination of the electric power system. It produces an audible noise based on a time series that is used to spot potential problems. A hybrid approach was suggested by [35] that employs the group method of data handling model for time series prediction and the wavelet energy coefficient for feature extraction. In comparison to LSTM and adaptive neuro-fuzzy inference systems, the proposed method demonstrated accuracy and was found to be considerably faster.

Three deep learning classification and regression models for fault region identification, classification, and location prediction were introduced by [36]. To model the spatiotemporal sequences of high-dimensional multivariate features and produce reliable classification and prediction results, deep sequential learning is used in conjunction with long short-term memory. Data for training and testing are gathered while various sorts of transmission line faults are introduced at various sites throughout various areas.

To find the best transformer model and locate various power system problems and unpredictable conditions, Thomas and K. V. [37] suggested a neural architecture search approach. To automatically construct ideal Transformer architectures with a lower search time cost, the authors applied the differential architecture search algorithm. The suggested fault analysis was performed using the VSB power line fault detection database and the industry-standard IEEE 14 bus distribution system. The real-world power line data for fault detection was used to examine the transferability of the suggested method’s architecture.

An overview and investigation of the fault prediction and fault location areas were presented in [38]. To achieve this, the methods and viewpoints currently in use in the context of fault prediction are first evaluated, followed by an analysis of fault location. In conventional distribution networks, smart grids, and microgrids, this paper examines numerous systems, their benefits and drawbacks, technical reports, and patents.

The diagnosis of power system faults using machine learning was thoroughly reviewed by [39]. The success of machine learning approaches was first attributed to attempts to include the problems with traditional fault diagnosis. Then, several fault diagnosis methods, including supervised and unsupervised learning approaches, were individually addressed. The benefits and drawbacks of each fault detection method were also covered, which will aid readers in choosing the best method for their own research.

Considering the high capabilities of the LSTM with the attention mechanism [40], in this paper, this model is used to forecast the increase in leakage current in contaminated insulators, as will be explained in detail in the next section. Besides the model employed, the considered dataset and the EWT technique used for signal preprocessing and denoising (noise reduction) will be explained. A summary of LSTM applied to forecasting works discussed in this chapter is presented in Table 1.

## 3. Methodology

LSTM with attention is designed to handle such complex patterns and dependencies. The attention mechanism in LSTM allows the model to focus on the most important inputs at each time step when making predictions. This can improve the model’s performance and stability compared to traditional LSTM models [41].

In the LSTM with attention applied to time series forecasting, the input data are typically divided into a series of time steps and processed through the LSTM. The attention mechanism then provides a weighted sum of the hidden states of the LSTM units, which is used as input to a fully connected layer for the final prediction [42].

### 3.1. Luong Attention Mechanism

The Luong attention mechanism is a method for calculating the attention weights in a neural network, including but not limited to the Seq2Seq model [43]. Given a set of hidden states $h_{1},h_{2},\ldots ,h_{n}$, the attention mechanism computes a set of attention weights $\alpha_{1},\alpha_{2},\ldots ,\alpha_{n}$ that represent the relative importance of each hidden state in creating the output [44]. Attention weights are calculated as follows:

(1) $$\begin{array}{c} \alpha_{t}=\frac{\exp(e_{t})}{\sum_{k=1}^{n}\exp\left(e_{k}\right)} \end{array}$$

where $e_{t}$ is a score that measures the compatibility between the target hidden state $h_{t}$ and the decoder state $s_{t-1}$ at the previous time step. The score is calculated using one of several functions, including dot-product, general, and concat. The dot-product function calculates the dot-product of the target hidden state and the decoder state, as follows:

(2) $$\begin{array}{c} e_{t}=h_{t}^{T}s_{t-1} \end{array}$$

The general function calculates the dot-product of the target hidden state and a linear transformation of the decoder state, as follows:

(3) $$\begin{array}{c} e_{t}=h_{t}^{T}W_{a}s_{t-1} \end{array}$$

where $W_{a}$ is a learned parameter. The concat function concatenates the target hidden state and the decoder state, and passes the concatenation through a feedforward neural network as follows:

(4) $$\begin{array}{c} e_{t}=v_{a}^{T}\tanh\left(W_{a}\left[h_{t};s_{t-1}\right]\right) \end{array}$$

where $v_{a}$ is a learned parameter and [;] denotes concatenation.

The attention weights are then used to compute a weighted sum of the hidden states, which is used as input to the decoder at the current time step. The weighted sum is computed as follows:

(5) $$\begin{array}{c} c_{t}=\sum_{k=1}^{n}\alpha_{k}h_{k}. \end{array}$$

Notice that the function used to determine the score ei depends on the job and available data. Typically, the dot-product function is employed when the dimensions of the hidden states are identical, and the general function is used when the dimensions are different. The concat function can capture more intricate interactions between the hidden and decoder states, but it needs to learn more parameters. A general overview of the method is given in Algorithm 1.
**Algorithm 1:** Luong Attention Mechanism
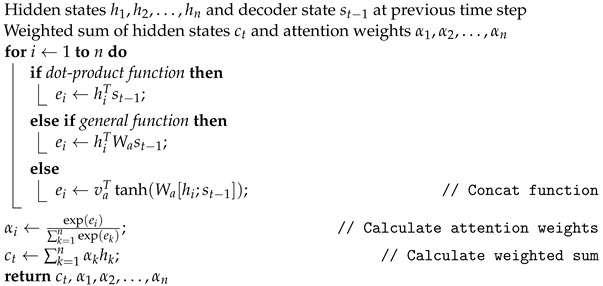


### 3.2. Encoder–Decoder LSTM

The encoder–decoder LSTM with attention is a form of neural network design that can be used for forecasting time series. In time series forecasting, the objective is to predict the future values of a time series using its historical values [45]. In this instance, the encoder LSTM analyzes the input time series and compresses it into the encoding, a vector of fixed length. The encoding serves as the network’s memory by summarizing the pertinent information in the time series. Let us denote the input time series as $x_{1},x_{2},\ldots ,x_{T}$, where *T* is the length of the time series [46]. The hidden state of the encoder LSTM at time step *t* is denoted as $h_{t}^{e}$. The encoding is calculated as the final hidden state of the encoder, $h^{e}=h_{T}^{e}$.

The decoder LSTM takes the encoding and generates the forecast, $y_{T+1},y_{T+2},\ldots ,y_{T+M}$, where *M* is the number of steps to forecast. The decoder LSTM also has an attention mechanism which allows it to focus on different parts of the encoding at each time step and generate the forecast one step at a time [47]. At time step *t*, the decoder computes the attention weights $\alpha_{1:T}$ over the encoding and calculates the context vector $c_{t}$ as a weighted sum of the encodings, as follows:

(6) $$\begin{array}{c} c_{t}=\sum_{k=1}^{T}\alpha_{k}h_{k}^{e} \end{array}$$

The decoder then updates its hidden state $s_{t}$ as a function of the previous hidden state $s_{t-1}$, the current input $y_{t-1}$, and the context vector $c_{t}$, as follows:

(7) $$\begin{array}{c} s_{t}=f\left(s_{t-1},y_{t-1},c_{t}\right) \end{array}$$

where *f* is the LSTM function. Finally, the decoder generates the next forecast $y_{T+t}$ by passing the hidden state $s_{t}$ through a fully connected layer with a linear activation function, as follows:

(8) $$\begin{array}{c} y_{T+t}=W_{o}s_{t}+b_{o} \end{array}$$

where $W_{o}$ and $b_{o}$ are learned parameters.

**Figure 1:**
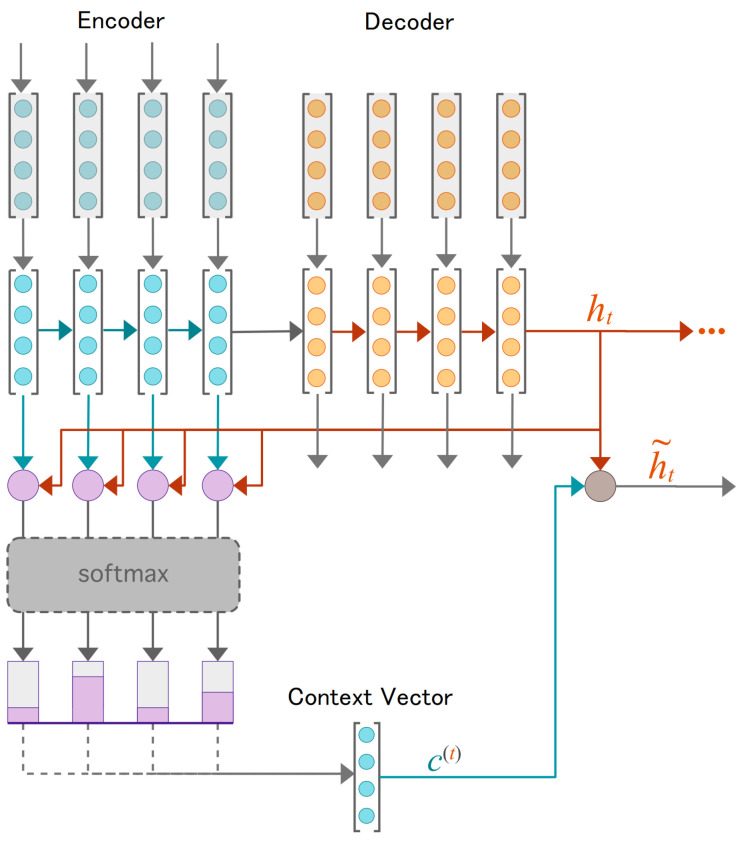
General overview of the network architecture, including a Seq2Seq encoder–decoder and attention mechanism.

The encoder–decoder LSTM with attention is trained to minimize a loss function, such as a mean squared error or mean absolute error, between the predicted and actual future values. The network generates the forecast step-by-step until the appropriate number of steps has been anticipated [48]. A general overview of the network architecture is shown in Figure 1.

### 3.3. Hypertuning

Selecting the ideal hyperparameters for a machine learning model’s optimal performance is a process known as hyperparameter tuning or hypertuning. It entails experimenting with hyperparameter combinations and evaluating their performance on a validation set. In this context, Optuna [49] is a Python module for hyperparameter optimization that provides multiple search algorithms for the hyperparameter space; the Tree-structured Parzen Estimator (TPE) algorithm is one of these algorithms [50].

TPE starts with a prior distribution over the hyperparameters, and at each iteration, it updates the prior and suggests new hyperparameters to try based on the model’s performance with the previous hyperparameters. TPE employs a probabilistic model to estimate the performance distribution of hyperparameters and to achieve a balance between exploration and exploitation in the search process [51].

The algorithm models the distribution of the performance of the hyperparameters using two probability density functions: l(x) for good hyperparameters (those that result in low loss values) and g(x) for bad hyperparameters (those that result in high loss values), where x is a hyperparameter configuration. The algorithm starts with a prior over these density functions and at each iteration, updates them based on the observed performance of the model to suggest the next hyperparameter configuration to try [52].

Let us denote the set of hyperparameters as x and the performance of the model with hyperparameters x as *y*. At iteration *t*, TPE first calculates the Expected Improvement (EI) function, which measures the trade-off between exploration and exploitation. The EI function is defined as:

(9) $$\begin{array}{c} EI\left(x\right)=\frac{l(x)}{g(x)} \end{array}$$

where l(x) and g(x) are the probability density functions for good and bad hyperparameters, respectively. Then, it suggests the next hyperparameter configuration to try as the one that maximizes the EI function:

(10) $$\begin{array}{c} x_{t+1}=\arg\max_{x}EI\left(x\right). \end{array}$$

Finally, the algorithm updates the probability density functions based on the observed performance of the model with the suggested hyperparameters:

(11) $$\begin{array}{cc} \; & l(x)\leftarrow \mathrm{updated}\;\mathrm{density}\;\mathrm{for}\;\mathrm{good}\;\mathrm{hyperparameters} \end{array}$$

(12) $$\begin{array}{cc} \; & g(x)\leftarrow \mathrm{updated}\;\mathrm{density}\;\mathrm{for}\;\mathrm{bad}\;\mathrm{hyperparameters} \end{array}$$

This procedure is repeated until a stopping requirement is satisfied, such as a maximum number of iterations or a minimum performance improvement [53]. In this paper, the rectified linear unit (ReLU), exponential linear unit (ELU), and hyperbolic tangent (Tanh) activation functions are evaluated.

### 3.4. Empirical Wavelet Transform

The Empirical Wavelet Transform (EWT) is an adaptive method for signal analysis, especially effective for time-varying signals. The procedure begins with signal preprocessing, denoted by x(t), where *t* represents time. In this phase, the signal is cleaned by removing any noise or trends that could affect the analysis.

The EWT process then assesses the signal’s frequency content and divides it into distinct subbands. These subbands are represented by a set of filters denoted as $\psi_{i}\left(\omega \right)$, where *i* ranges between 1 and *N* and ω is the angular frequency. The filters are designed based on the characteristics of the signal, ensuring that they cover its entire frequency spectrum. Once the filters have been designed, the EWT applies them to the signal in order to determine the wavelet coefficients $c_{i}\left(t\right)$ for each filter [54,55].

## 4. Experiments and Results

In this section, the results of the evaluation of the proposed model are presented. Initially, the influence of the data initialization is analyzed, explaining how the Seq2Seq dataset was used. Then, the model is optimized with Optuna, the EWT filter is used for noise reduction, and statistical analysis is presented. Finally, benchmarking is performed. The best overall results from this section are highlighted in bold and the best results from each comparison are underlined, with exception of the statistical analysis of the dataset.

### 4.1. Dataset

The experiments were performed in a saline chamber, using an applied voltage of 8.66 kV (RMS 60 Hz). The contamination was gradually increased until a flashover occurred following the NBR 10621 (equivalent to IEC 60507) standard. The NBR 10621 defines the characteristics of supportability under contamination for insulators in power grids. The experiment was conducted under controlled conditions (humidity, temperature, pressure, and contamination). The statistical characteristics of the dataset are presented in Table 2. The mean, median, mode, range, variance, standard deviation (Std. Dev.), percentile (%ile), interquartile range (IQR), skewness, and kurtosis are considered.

**Figure 2:**
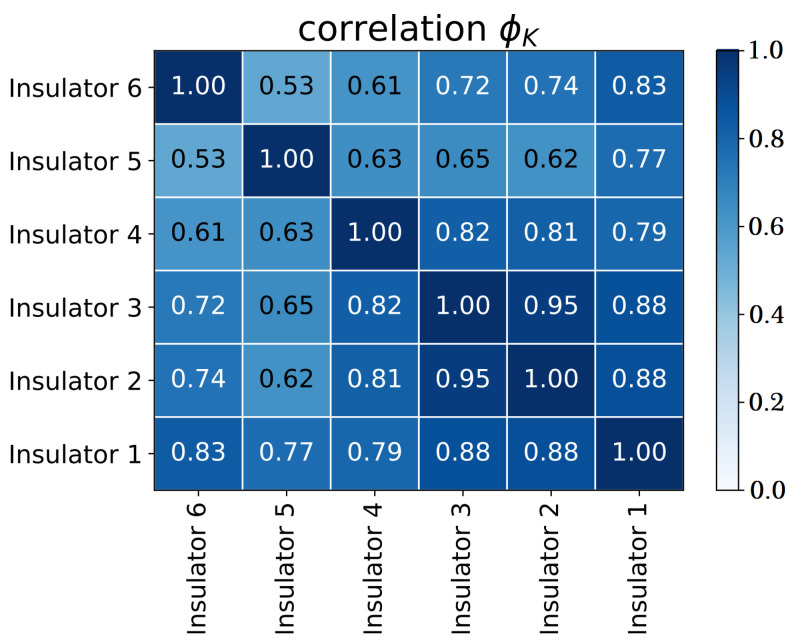
Correlation of the samples based on their leakage current values.

In the salt chamber, six insulators (15 kV class) were evaluated, and two were selected according to the phik correlation [56] between the recorded leakage current values. This relationship is presented in Figure 2.

**Figure 3:**
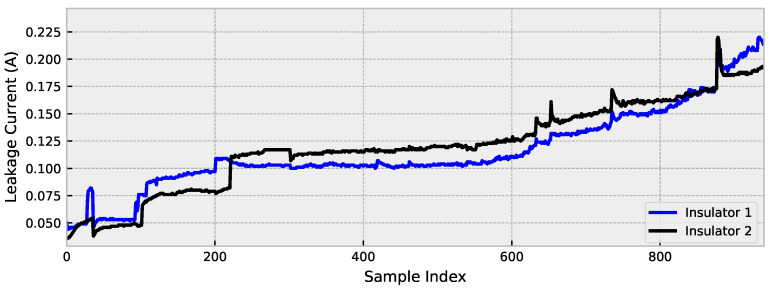
Leakage current of the evaluated insulators.

The highest correlation between samples occurs between insulators 2 and 3, with 98% correction, and for this reason, these insulators were considered in this paper. In these insulators, the flashover occurred after 26.11 h of running the experiment. The records were taken every second, and thus there were 9.4 ×$10^{4}$ records. To proceed with the evaluation, a downsample of order 10 was applied to reduce the computational complexity, and therefore values up to 940 records are evaluated (see Figure 3).

### 4.2. Experiment Setup

The algorithm was written in Python using the *keras* framework and *statsmodels*, the experiments were evaluated in Google Colaboratory (Colab), using a graphic processing unit NVIDIA Tesla T4, a central processing unit Intel(R) Xeon(R) @2.30GHz, and 12GB of random access memory.

To ensure that overfitting does not occur, the early stop criterion was used, which stops training when there is no improvement in the model during training. This procedure was based on the mean square error (MSE). For a comparative analysis of the models’ performances, the MSE, mean absolute error (MAE), and mean absolute percentage error (MAPE) were evaluated, given by:

(13) $$\mathrm{MSE}=\frac{1}{n}\sum_{i=1}^{n}{\left(y_{i}-{\hat{y}}_{i}\right)}^{2}$$

(14) $$\mathrm{MAE}=\frac{1}{n}\sum_{i=1}^{n}\left(y_{i}-{\hat{y}}_{i}\right)$$

(15) $$\mathrm{MAPE}=\frac{1}{n}\sum_{i=1}^{n}\left|\frac{y_{i}-{\hat{y}}_{i}}{y_{i}}\right|\times 100$$

where *n* is the length of the signal, $y_{i}$ is the observed value, and ${\hat{y}}_{i}$ is the predicted output.

For the final comparative analysis, the stacking ensemble learning method is evaluated. The linear, radial basis function (RBF), and polynomial kernel functions were considered using a support vector regression (SVR). Additionally, the quadratic programming (L1QP), iterative single data algorithm optimization (ISDA), and sequential minimal optimization (SMO) solver are compared.

### 4.3. Data Initialization

The way that the data are initialized might have consequences on the model’s performance, and thus, with a bigger batch size, the model tends to be faster, since it loads more data in a single step, and the percentage of data used for training can also have significant results on the model’s performance, considering that a larger number of data for training can make it have better performance. Table 3 shows a comparison between the percentage ratio used for training and testing the model, as well as the batch size variation.

All results using a larger batch size were faster to converge, however, in this research, the objective was to have a model with lower error and not one that simply faster; hence, this criterion was not considered in defining the best way to load the data. The lowest MSE and MAE were found using 90% of the data for training, showing that a larger dataset can improve the model’s trainability.

Since the focus of this research was to evaluate the use of fewer data for training to achieve a greater prediction horizon, 70% of the data were considered for training, with this focus the best result in error reduction occurred using a batch size equal to 16 (regarding a lower MSE, MAE, and MAPE); for this reason, this setup was defined as the default and further analyses were based on this definition.

### 4.4. Denoising

**Figure 4:**
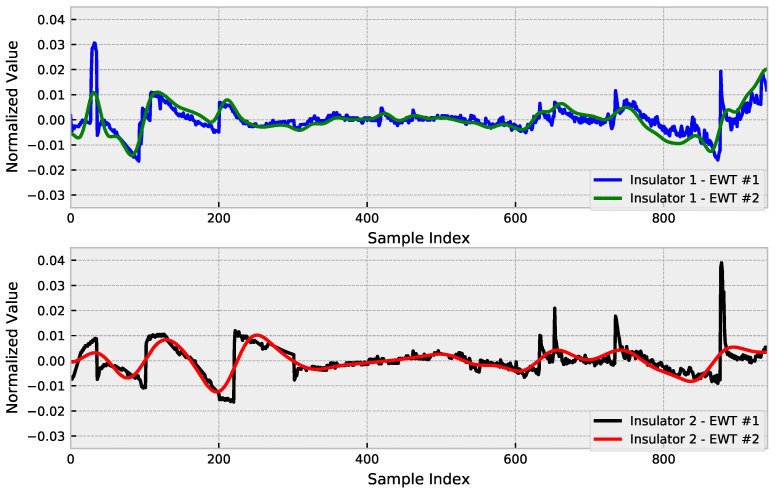
EWT detrend results.

To perform noise reduction, EWT was applied to the preprocessed leakage current signals, considering that the detrend is used as a co-feature of the network, this being an already filtered input signal to support training. The results of using EWT over the original detrend are presented in Figure 4.

The noise that occurs in the measurements is due to the nonlinearity of the leakage current. One of the reasons for the nonlinearity of the leakage current in contaminated insulators is that pollution does not deposit linearly on the surface, and there are variations in pressure and humidity caused by the discharge. Therefore, these variations can be disregarded when the analysis is performed over a time series.

In the context of predicting leakage current, which is an indication that a further failure is likely to occur possibly due to an increase in the surface conductivity of the insulator, reducing the variations that are not a component of the increasing trend is promising, since this does not affect the focus of the assessment and reduces the complexity of the analysis.

### 4.5. Hyperparameter Optimization

**Figure 5:**
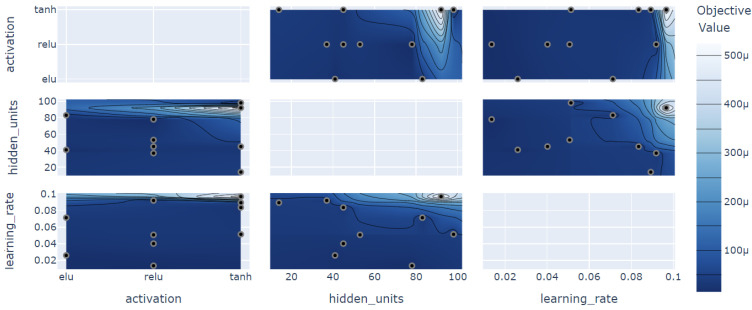
Hyperparameter optimization with Optuna.

To obtain an optimized model that has a better performance for solving the problem addressed in this paper, there is hypertuning, which defines the optimal hyperparameters to be used in the model. For hyperparameter optimization, Optuna was used, which is a specific framework for it. The use of 10–100 hidden units, the activation functions ReLU, ELU, Tanh, and the learning rate from 0.001–0.1 were evaluated, the results are presented in Figure 5.

The optimization of the hyperparameters returned the value of 78 hidden units, the ReLU activation function, and the learning rate of 1.36 ×$10^{-2}$. These values were used for all experiments for a fair evaluation. In this comparative analysis, the most important hyperparameter for model optimization was the learning rate, which has an importance of 89.22% to achieve the objective result of performance optimization, whilst the hidden units had an importance of 10.71% and the activation function had only 0.07%, this being the least important variation in the model optimization.

**Figure 6:**
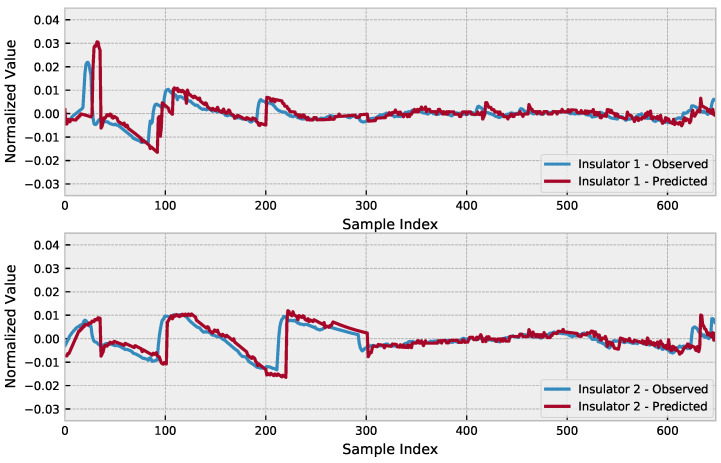
Prediction results compared to the observed signal.

Including co-features can aid in capturing additional information that may not be included in the time series itself, or even guide the prediction in a trend as in the proposed model. Relying only on the denoised signal could lead to missing important behaviors in the original signal; thus, we opted to input both signals for both insulators as a way to provide the model with enough information to keep the peculiarities of each insulator, while trying to follow the trend based on the correlation existing between the signals. Assuming the optimized hyperparameters, the use of the attention mechanism in the LSTM, and using the EWT co-features, the prediction results are presented in Figure 6.

### 4.6. Benchmarking

In this subsection, the proposed optimized EWT-Seq2Seq-LSTM with the attention method is compared to other models considering the characteristic of a Seq2Seq signal. The results are presented in Table 4. The difference between the compared models is that the standard model does not use the attention mechanism, EWT-Seq2Seq-LSTM with attention is not optimized, and the proposed model uses hyperparameter optimization besides the attention mechanism. For a fair comparison, all models use the same EWT-based input signal, which means all signals are preprocessed.

An important observation is that hyperparameter optimization can make the model converge considerably faster; therefore, special attention needs to be given to the early stop. If the early stop has a weakly constrained criterion (with a reduced number of unimproved epochs to stop the training), there might be an early stop precipitated.

The proposed optimized EWT-Seq2Seq-LSTM with attention was shown to have an improvement in all error metrics evaluated, this being faster than the model without optimization, and although the standard model is quicker to converge, its inferior results regarding error do not justify its use. Based on these results, a statistical analysis was performed to evaluate the reliability of the model over several experiments.

For comparative purposes, Table 5 presents the analysis using the stacking ensemble learning method considering the variation of solvers and the kernel functions. It can be observed that the MSE error values are higher using the ensemble method, but only the MAPE was lower in the comparative analyses.

Other analyses using equivalent signals can be found in the work of Sopelsa Neto et al. [4]. One difference that should be noted, compared with our work, is that Sopelsa Neto et al. [4] did not use the MAPE in percent to present the results. Comparatively, all the MSE results were lower than the proposed optimized EWT-Seq2Seq-LSTM with the attention model. This shows that the proposed model is superior to other methods even when the wavelet transform is used.

### 4.7. Statistical Assessment of the Proposed Method

**Figure 7:**
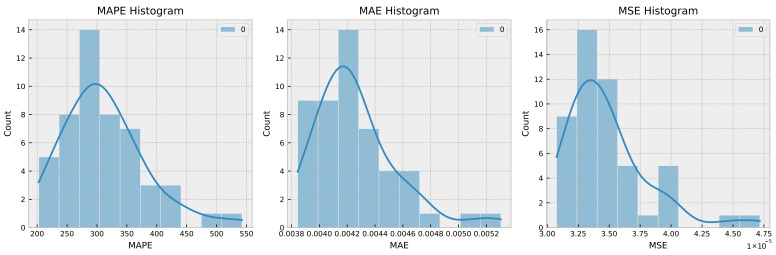
Histogram of MSE, MAE, and MAPE for 50 runs with Gaussian noise.

**Figure 8:**
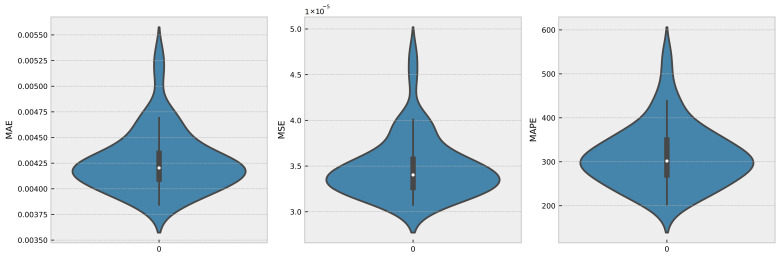
Violin of MSE, MAE, and MAPE for 50 runs with Gaussian noise.

To evaluate the robustness of a time series model, it is typical to train the model numerous times and compare its performance measures between runs. This can help discover any potential sources of variability in the model’s performance, such as random parameter initialization or changes in the training data utilized for each run. In this instance, the proposed model was trained 50 times, with Gaussian noise being introduced in each run, and the MSE, MAE, and MAPE for each run were recorded. Figure 7 and Figure 8 show, respectively, the histogram of the boxplot of the trained models.

From the histogram and boxplot, it is clear that the model’s variance remains within acceptable limits. This indicates that the model is resilient and capable of performing well across a broad range of training data and parameter settings. In addition, the boxplot reveals that the median performance measure values are consistent across all 50 training runs with random Gaussian noise, further supporting the robustness of the model.

## 5. Final Remarks and Conclusions

The increase in leakage current is related to the increase in surface conductivity, which occurs in distribution power grid insulators due to the accumulation of contaminants on their surface. When the leakage current becomes considerably high, flashovers occur, resulting in power supply shutdowns. The prediction that the leakage current increases is an indication that a discharge will occur and can therefore be used to evaluate the influence of the environment on the performance of insulators over time.

There are several techniques that can be used to enhance a prediction model, such as the empirical wavelet transform for noise reduction, the attention mechanism to improve the predictive ability of the model, and the optimization of its hyperparameters. All these techniques are combined in this paper to obtain the proposed optimized EWT-Seq2Seq-LSTM with attention which proves to be superior in comparative analysis.

The proposed model optimized EWT-Seq2Seq-LSTM with attention had an MSE of 1.06 ×$10^{-5}$, which was superior to the standard LSTM and the model without using Optuna hypertuning, showing that it is promising to use the attention mechanism and the optimization of the network hyperparameters.

Future work can be conducted to evaluate uncorrelated leakage current and determine how well the model performs when uncorrelated data are used for training. The leakage current can also be evaluated in other components of the electrical distribution system, and it is promising to evaluate the insulators manufactured from polymeric materials, which are increasingly being used.

## Figures and Tables

**Table 1:** Summary of LSTM for time series forecasting.

Author(s)	Methodology
Zang et al. [25]	LSTM with a self-attention mechanism for day-ahead residential load forecasting.
Qu et al. [26]	Attention-based LSTM model for short-term prediction.
Fazlipour et al. [27]	LSTM-based stackable autoencoder with attention mechanism for short-term load forecasting.
Lin et al. [28]	Dual-attention LSTM model for short-term load forecasting with probabilistic predictions.
Zhu et al. [29]	Dual-attention LSTM model for analyzing characteristics of daily peak load simultaneously.
Li et al. [30]	Deep learning-based interval prediction model combining attention mechanism and LSTM.
Meng et al. [31]	Attention mechanism for LSTM forecasting model with empirical wavelet transform % for electricity price prediction.
Qin et al. [32]	Multi-task LSTM model with attention mechanism for predicting loads of a substation.
Dai et al. [33]	Combined LSTM with attention mechanism and XGBoost for short-term load forecasting.

**Table 2:** Statistical characteristics of the dataset.

	Insul. 1	Insul. 2	Insul. 3	Insul. 4	Insul. 5	Insul. 6
Mean	0.08947	0.11890	0.12323	0.06242	0.02737	0.04913
Median	0.13200	0.10400	0.11800	0.09500	0.03800	0.05000
Mode	0.00000	0.10300	0.11700	0.00000	0.00000	0.00000
Range	0.26400	0.22700	0.26600	0.19300	0.17100	0.75500
Variance	0.00594	0.00173	0.00186	0.00216	0.00058	0.00261
Std. Dev.	0.07710	0.04156	0.04309	0.04643	0.02403	0.05113
25th %ile	0.00000	0.10200	0.11200	0.00000	0.00000	0.00000
50th %ile	0.13200	0.10400	0.11800	0.09500	0.03800	0.05000
75th %ile	0.13800	0.13800	0.15300	0.10100	0.04200	0.09000
IQR	0.13800	0.03600	0.04100	0.10100	0.04200	0.09000
Skewness	0.20900	0.88310	0.17629	−0.31889	0.25156	2.92524
Kurtosis	−1.05212	0.84858	0.36154	−1.45492	0.20065	36.47633

**Table 3:** Assessment of the influence of how the dataset is loaded.

Train/Test (%)	Batch Size	MSE	MAE	MAPE	Time (s)
70/30	8	$1.55\times 10^{-5}$	$2.75\times 10^{-3}$	$2.04\times 10^{2}$	1117.92
	16	$1.48\times 10^{-5}$	$2.40\times 10^{-3}$	**1.50 × $10^{2}$**	330.82
	32	$1.57\times 10^{-5}$	$2.69\times 10^{-3}$	$1.91\times 10^{2}$	148.29
	64	$1.65\times 10^{-5}$	$2.71\times 10^{-3}$	$1.93\times 10^{2}$	90.23
80/20	8	$1.38\times 10^{-5}$	$2.50\times 10^{-3}$	$2.33\times 10^{2}$	628.88
	16	$1.34\times 10^{-5}$	$2.31\times 10^{-3}$	$2.48\times 10^{2}$	281.13
	32	$1.38\times 10^{-5}$	$2.63\times 10^{-3}$	$3.18\times 10^{2}$	208.49
	64	$1.50\times 10^{-5}$	$2.73\times 10^{-3}$	$3.60\times 10^{2}$	**60.34**
90/10	8	$1.74\times 10^{-5}$	$2.99\times 10^{-3}$	$2.95\times 10^{2}$	679.75
	16	$1.59\times 10^{-5}$	$2.74\times 10^{-3}$	$3.66\times 10^{2}$	352.54
	32	**1.17 × $10^{-5}$**	**2.16 × $10^{-3}$**	2.44 × $10^{2}$	208.50
	64	$1.61\times 10^{-5}$	$2.87\times 10^{-3}$	$3.73\times 10^{2}$	148.19

**Table 4:** Model comparison analysis.

Model	MSE	MAE	MAPE	Time (s)
EWT-Seq2Seq-LSTM Standard	1.18 × $10^{-5}$	$2.32\times 10^{-3}$	$2.31\times 10^{2}$	**239.89**
EWT-Seq2Seq-LSTM with Attention	$1.12\times 10^{-5}$	$2.26\times 10^{-3}$	$2.33\times 10^{2}$	328.68
Proposed Method	**1.06× $10^{-5}$**	**2.08 × $10^{-3}$**	**2.11× $10^{2}$**	277.59

**Table 5:** Evaluation of stacking ensemble learning method.

Solver	Function	MSE	MAE	MAPE	Time (s)
L1QP	Linear	1.50$\times 10^{-3}$	2.99$\times 10^{-2}$	1.67$\times 10^{1}$	3.59
	RBF	$6.25\times 10^{-3}$	$7.01\times 10^{-2}$	$4.11\times 10^{1}$	1.69
	Polynomial	$2.04\times 10^{-1}$	$1.71\times 10^{-1}$	$8.79\times 10^{1}$	1.67
ISDA	Linear	1.48$\times 10^{-3}$	2.98$\times 10^{-2}$	1.66$\times 10^{1}$	1.38
	RBF	$4.91\times 10^{-3}$	$6.11\times 10^{-2}$	$3.56\times 10^{1}$	0.53
	Polynomial	$4.35\times 10^{-1}$	$3.25\times 10^{-1}$	$1.65\times 10^{2}$	18.45
SMO	Linear	**1.45$\times 10^{-3}$**	**2.94$\times 10^{-2}$**	**1.65$\times 10^{1}$**	0.92
	RBF	$6.25\times 10^{-3}$	$7.01\times 10^{-2}$	$4.12\times 10^{1}$	**0.47**
	Polynomial	$2.59\times 10^{-1}$	$1.77\times 10^{-1}$	$9.05\times 10^{1}$	32.03

## Data Availability

For future comparisons, the proposed method and the considered dataset are available at: https://github.com/SFStefenon/EWT-Seq2Seq-LSTM-Attention (accessed on 10 March 2023).
